# Faircare: A new direction for Health Care and Policy in Ireland

**Published:** 2009-05-23

**Authors:** James Reilly

**Figure:**
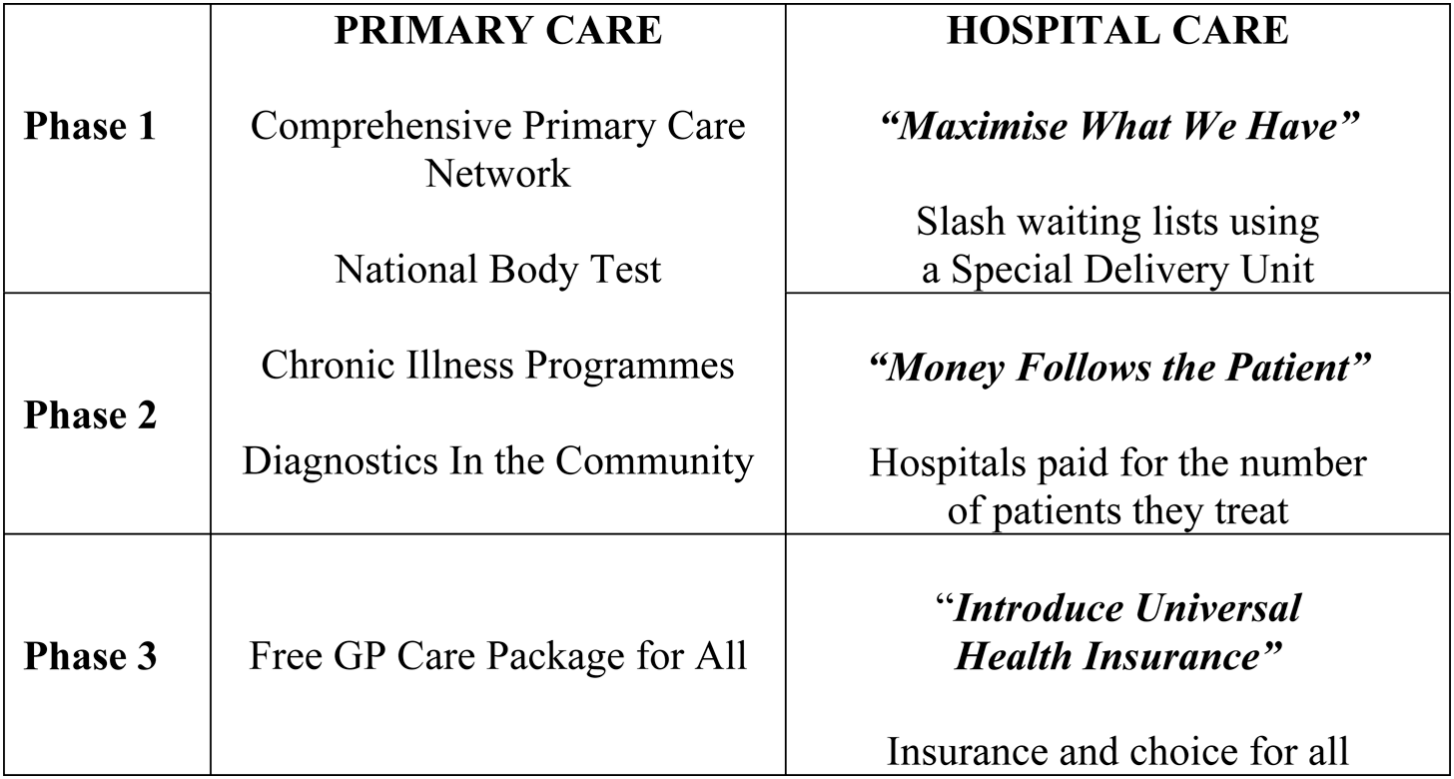


The quadrupling of the Irish health budget since 1997 has proved one thing - a poorly organised and managed health system cannot be fixed with money alone.

Ireland is spending close to the EU average on health, on a per capita basis, but Irish people are receiving a level of service well below the EU average. Ireland’s health service is ranked 15^th^ in Europe for quality and 24^th^ in terms of value for money.

Our health system is broken. For the last ten years the Government has thrown money at every problem, without making any fundamental change to the way the health system works. Even in the midst of economic crisis, it still refuses to make significant reform. Billions are wasted, waiting lists increase, and the sick and infirm pay the price for the Government’s incompetence.

Fine Gael’s ***“FairCare”*** proposals, by contrast, represent the most fundamental reform of the health system since the formation of the State. We will abolish long-term waits on trolleys in A&E, slash waiting lists in hospitals, and eliminate the unfair and inefficient public/private divide by introducing Universal Health Insurance (UHI). We will also reform the Primary Care system to ensure that more patients are treated safely outside hospitals by their GPs.

## Primary Care Reform is Crucial

Since it was first announced in 2001, the Government has consistently failed to meet the key targets in its Primary Care strategy.

Fine Gael will give Primary Care the priority it deserves. By the end of our first term in Government, we will have a comprehensive network of new Primary Care centres to serve our communities. The community they serve will determine the size and scale of the centre. Depending on population coverage, their services will include X-Ray, Ultrasound, Endoscopy, Physio, CT and MRI scanning, etc. The centres will also include rooms for visiting specialists and will accommodate a robust community mental health service.

Patient flows to hospital will be further reduced by the availability of a National Body Test (NBT) to pick up illness early, and by the existence of chronic illness programmes for diseases such as high blood pressure, asthma, etc. to prevent the complications that land people in hospital.

We believe that the capital costs of this programme can largely be borne by the private sector, if appropriate long-term contracts are put in place. If additional incentives are required, such as Accelerated Capital Depreciation, this requirement will be addressed.

## A 3-Phase Programme

Reforming the Irish health system will not be easy. The last thing Ireland needs is another ill-conceived experiment like the formation of the Health Service Executive (HSE). In addition, any reform must be undertaken within Fine Gael’s overall budgetary framework. We have, therefore, divided our FairCare programme into three distinct, but over-lapping phases that will allow us to gradually introduce reforms in a way that is both carefully planned and affordable.

### Phase 1: Maximise What We Have (Implemented from Year 1)

In the first phase of FairCare, we will change the way hospitals work and, as indicated above, will also significantly strengthen Ireland’s Primary Care system.

As part of our hospital reform programme, we will make the Minister of Health directly responsible for hitting key targets. Progress will be measured daily by real time information systems, and a Special Delivery Unit will be established to assist the Minister. A similar unit was successfully used in Northern Ireland to help slash waiting lists, e.g., inpatient waiting lists for those waiting more than 3 months fell by 80% from 2004 to 2008. Crucially, this was done without significantly increasing spending,

Fine Gael recognises that significant bed capacity in hospitals could also be freed-up if patients facing delayed discharge or requiring rehabilitation could be treated in the Community. We will publish specific proposals on this issue over the next few months to address the current deficits in long-term care and rehabilitation.

Fine Gael will also ensure that resources, arising from the sale of psychiatric institutions and lands, will be ring-fenced to mental health. Psychiatric illness must be treated like any other illness, and resourced accordingly.

### Phase 2: Introduce “Money Follows the Patient” (Year 3)

Under the current system of fixed budgets, each additional patient is effectively a “cost” to the health service. This system provides no incentives for efficiency or productivity. Under MFTP, health providers will be paid for how many patients they treat. Patients will be a source of “income” rather than a “cost”, just as they are in private hospitals today.

MFTP will mean that decision-making is increasingly devolved to the hospitals themselves. Once MFTP is introduced, the National Treatment Purchase Fund will be closed, saving around €100 mn a year. Long term, we expect MFTP to increase efficiency by as much as 10%.

### Phase 3: “Universal Health Insurance” (Implemented in Year 5)

Once the first and second phases of FairCare have been successfully implemented, Fine Gael will introduce Universal Health Insurance (UHI), a system that is widely used in Europe and in Canada. UHI will only be introduced once waiting lists have been significantly reduced in Phases 1 and 2. In the interim, the current system of voluntary insurance in Ireland will remain in place.

Within its first 30 days in office, a Fine Gael Government will establish a **UHI COMMISSION**, which will include representatives from all of the major stakeholders in the health service. Its primary task will be to build a consensus around the practical measures that need to be taken to prepare the health system for UHI. One of the keys to success for any insurance system is strong regulation. The Regulator will be answerable to the Minister and the Oireachtas.

Fine Gael proposes to introduce the Dutch model of UHI in Ireland, with mandatory health insurance for everyone, to be chosen from a selection of providers. The Netherlands spends only slightly more than us on health on a per capita basis, but is ranked number 1 in Europe for quality and Number 2 for value for money (Source: European Health Consumer Index 2008).

The Dutch system of UHI has strict community rating and an obligation to cover, which means that insurance companies will not be able to discriminate against anybody on the basis of age, sex, medical history, etc. This will be underpinned by a system of Risk Equalisation, which will compensate insurers for covering higher risk, higher cost patients. The insurance model will also address mental health.

Under UHI, everyone will receive a package of free GP care, paid for by some rebalancing of the tax system, and significant savings as the insurance companies bring down costs. There will, in addition, be significant savings in administration. At the moment, Ireland has two administrative systems for health – one public (the HSE) and one private (the insurance companies) – resulting in enormous duplication and waste. Over time, these two systems will become one, run by the insurance companies. As a result, the number of administrative staff employed in the HSE will likely fall by at least 5,000, as its role becomes more focused on long term care, public health, etc.

UHI will require the insurance industry in Ireland to play a much greater role in negotiating contracts with hospitals and other providers, and in driving innovation, than has been the case to date. Fine Gael will not introduce UHI until it is certain that the insurance companies are capable of taking on the expanded role required of them. A Fine Gael Government will encourage insurance companies from other European countries, who have experience of implementing social insurance models, to enter the Irish market.

**Figure 1:**
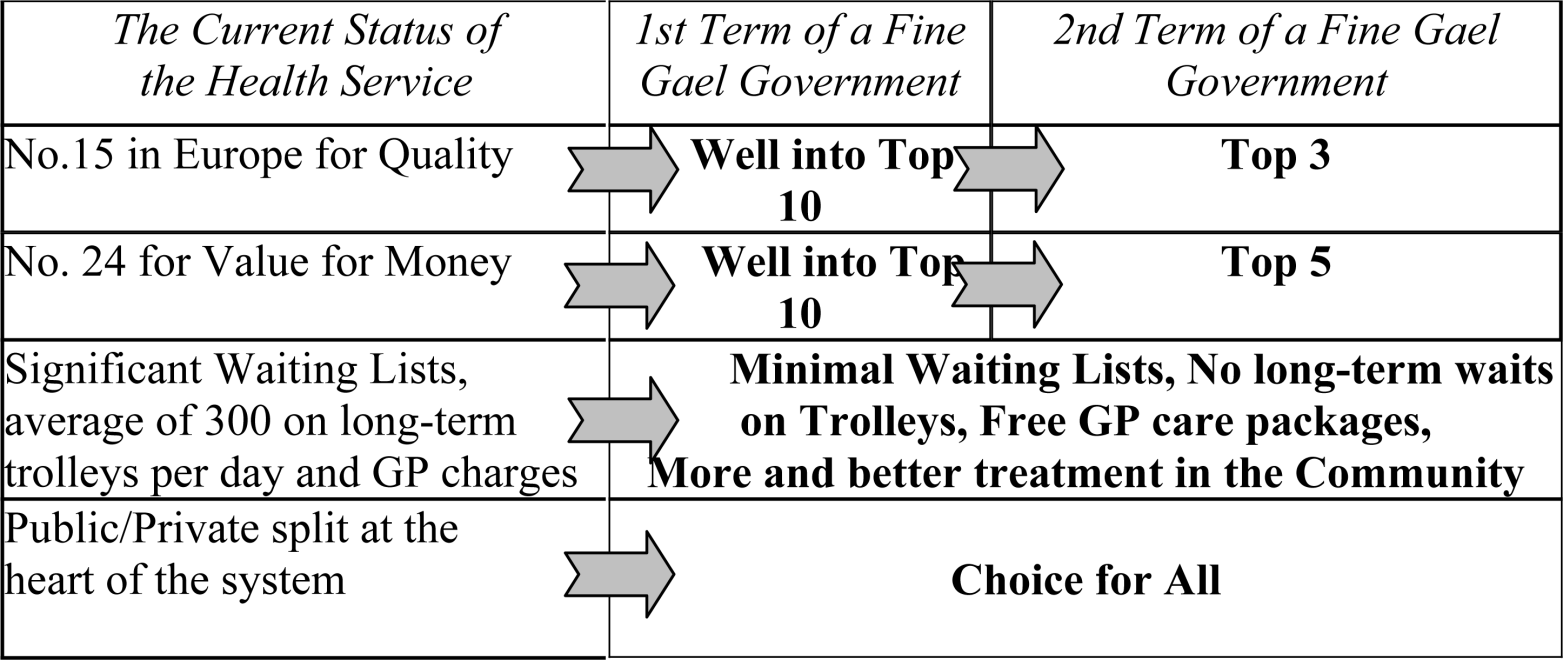
Making Ireland’s Health Service One of the Best in Europe

Figure 1 summarises Fine Gael’s ambitious but achievable targets for all three phases of FairCare. Our goal is to transform the Irish health service into one of the best in Europe, and create a health system that we can all be proud of.

## Five Key Principles of FairCare

Fine Gael’s approach to healthcare reform is essentially pragmatic. We are not bound by ideology or dogma, but will apply to Ireland best practice from other successful health systems. However, FairCare is underpinned by five key principles:

### 1. ACCESS is a right - not a privilege

In health, delayed treatment can lead to pain, complications and even death. More than 150,000 people are currently waiting for an outpatient appointment, some for up to 8 years, and 40,000 people are on the inpatient waiting list. Each day, an average of more than 300 people have long-term waits on trolleys. These delays are unfair and some may well prove fatal.

### 2. TRANSPARENCY and EFFICIENCY are not optional extras

The Government seems to believe that efficiency is somehow an optional extra. It is no surprise, therefore, to find Ireland ranked 24^th^ in Europe for value for money. FairCare, by making the system much more efficient and transparent, will allow more patients to be treated, and help ensure that taxpayers’ money is not wasted.

### 3. FAIRNESS: Equal treatment for equal need

The two-tier health system undermines SOLIDARITY within society, and encourages duplication and waste by creating two administrative systems. However, any move to abolish peoples’ right to health insurance would reduce choice. The fairest solution is to ensure that everyone has health insurance.

### 4. CHOICE for all

Fine Gael is not proposing the abolition of private health insurance. We are proposing that Ireland ultimately moves to a single-tier UHI system, where everyone has mandatory health insurance and can choose from a range of insurance plans. **In other words, we want to give choice to everyone.**

### 5. ACCOUNTABILITY: The buck stops where?

The HSE has allowed the Minister of Health and her Department to distance themselves from responsibility for the health service. This has created a huge democratic deficit and removed vital leadership. Fine Gael will place responsibility for the health service back where it belongs – with the Minister.

## More Detailed Reports Will be Published Over the Next Few Months

The FairCare proposals included in this document provide the road map for radically changing the Irish health system. They are not intended to address every aspect of health policy. Over the next few months, we will publish additional, more detailed reports on key aspects of the health system, such as the role of local hospitals and the future of care in Ireland. All constructive comments and proposals on how we might further strengthen FairCare will be gladly received.

